# Clinical Unmet Needs in the Treatment of Adrenal Crisis: Importance of the Patient’s Perspective

**DOI:** 10.3389/fendo.2021.701365

**Published:** 2021-07-20

**Authors:** Kim M. J. A. Claessen, Cornelie D. Andela, Nienke R. Biermasz, Alberto M. Pereira

**Affiliations:** Department of Medicine, Division of Endocrinology, and Center for Endocrine Tumors Leiden (CETL), and European Reference Network on Rare Endocrine Conditions (Endo-ERN), Leiden University Medical Center, Leiden, Netherlands

**Keywords:** adrenal insufficiency, cortisol, adrenal crisis, quality of life, mortality, patient’s perspective, unmet needs, hydrocortisone replacement

## Abstract

Adrenal crisis is the most severe manifestation of adrenal insufficiency (AI), but AI can present with variable signs and symptoms of gradual severity. Despite current hormone replacement strategies, adrenal crisis is still one of the leading causes of mortality in AI patients. Although underlying factors explaining differences in interindividual susceptibility are not completely understood, several subgroups are particularly vulnerable to adrenal crises, such as patients with primary AI, and patients treated for Cushing’s syndrome. Currently, the health care professional faces several challenges in the care for AI patients, including the lack of reliable biomarkers measuring tissue cortisol concentrations, absence of a universally used definition for adrenal crisis, and lack of clinical tools to identify individual patients at increased risk. Also from the patient’s perspective, there are a number of steps to be taken in order to increase and evaluate self-management skills and, finally, improve health-related quality of life (HR-QoL). In this respect, the fact that inadequate handling of AI patients during stressful situations is a direct consequence of not remembering how to act due to severe weakness and cognitive dysfunction in the context of the adrenal crisis is quite underexposed. In this narrative review, we give an overview of different clinical aspects of adrenal crisis, and discuss challenges and unmet needs in the management of AI and the adrenal crisis from both the doctor’s and patient’s perspective. For the latter, we use original focus group data. Integration of doctor’s and patient’s perspectives is key for successful improvement of HR-QoL in patients with AI.

## Highlights

- An adrenal crisis is the most severe manifestation of AI, resulting in a range of signs and symptoms.
- An adrenal crisis remains one of the leading causes of mortality in AI patients despite chronic hormone replacement.
- Several subgroups of AI patients are increasingly vulnerable to an adrenal crisis, including patients with primary AI, a history of Cushing’s syndrome and the use of mitotane for metastatic adrenocortical carcinoma.
- The absence of a universally used definition of adrenal crisis leads to a delay in diagnosis and initiation of treatment, but also results in uncertainty from the patient’s perspective.
- Imperfections in current hormone replacement strategies and lack of reliable biomarkers to measure cortisol levels on tissue level complicate establishment of the optimal replacement dose for the individual patient.
- Evidence on the effectiveness of different patient education strategies is scarce, but there is a clear need for further improvement of education of patients and their family, as is well illustrated by different statements from the focus group, including “*Learning how to inject is also important for my partner, such as what exactly happens during a crisis and what exactly does a crisis entail*”.
- Integration of the doctor’s and patient’s perspective is the key to improve HR-QoL in AI patients, which can only be achieved by close collaboration of all health care professionals, including patient associations.

## Introduction

Adrenal insufficiency (AI) is a serious condition in which patients suffer from insufficient cortisol production, requiring lifelong hormone replacement. Causes of AI can be classified into primary, secondary, and glucocorticoid-induced or tertiary causes based on underlying pathology (1). In short, in primary AI, also called Addison’s disease, the adrenal gland itself is directly affected, mostly by destructive autoimmunity or innate error of steroidogenesis resulting in deficiency of both glucocorticoids and mineralocorticoids. The secondary form of AI is caused by insufficient adrenocorticotropin hormone (ACTH) production of the pituitary, which can be isolated or part of multiple pituitary hormone deficiencies. Tertiary AI is the consequence of suppressed hypothalamic corticotrophin-releasing hormone (CRH) release, due to long-lasting exogenous steroid use in most patients (1).

The initial clinical presentation of AI can widely vary from (often mild) non-specific physical, cognitive and psychological complaints to the potentially fatal adrenal crisis requiring immediate treatment. Due to the gradual onset and non-specificity of initial symptoms, AI is a typical condition with a delayed diagnosis and a high rate of initial misdiagnosis (2, 3). Although hormone replacement therapy results in improvement of symptoms in most patients, chronically treated patients report persistent morbidity and impairments in health-related quality of life (HR-QoL) (4–8). It is plausible that the impairments in HR-QoL are, at least partly, caused by intrinsic imperfections in hydrocortisone replacement strategies (5, 9). Since current replacement strategies cannot mimic physiological hormonal secretion patterns, and, since many factors affect an individual’s glucocorticoid sensitivity, it remains difficult to establish the optimal replacement dose for the individual patient (10, 11), especially since there are currently no biomarkers that accurately reflect tissue cortisol concentrations (12). Furthermore, patients need to make glucocorticoid dose adjustments themselves in case of (upcoming) stressful events, relying on the self-care skills of patients. Despite individualized treatment strategies with dose adjustments in stressful situations, premature mortality in AI patients is still increased compared to the general population, with adrenal crisis among the leading causes of death (10%), next to cardiovascular disease, malignancies and infections (3, 13–17).

Given the broad continuum of clinical symptoms of adrenal crisis, its potential fatal character, and its significant negative impact on HR-QoL, the aim of this narrative review is to discuss adrenal crisis in more detail. Furthermore, challenges and unmet needs in the management of AI and prevention of adrenal crisis will be discussed from the perspective of the doctor, as well as from the perspective of the patient. For further illustration of the patient’s view, original focus group data will be presented. Finally, we will discuss future innovative perspectives with focus on integration of both doctor’s and patient’s perspective to further optimize the management of AI and prevention of adrenal crisis, with the ultimate goal improvement in HR-QoL.

## Adrenal Crisis: The Most Serious and Life-Threatening Manifestation of Adrenal Insufficiency

Here, we will zoom in on different aspects of adrenal crisis: the most serious manifestation of AI. Adrenal crises are life-threatening conditions due to the occurrence of acute absolute or relative glucocorticoid deficiency, being a real and constant danger to all AI patients with substantial impairment of HR-QoL. Moreover, despite hydrocortisone replacement, adrenal crisis is still one of the leading causes of death in AI patients (3), with an associated mortality rate of 0.5 per 100 patient-years (PY) (15). Mortality rates are even higher among patients with previously undiagnosed hypoadrenalism and in primary AI. However, since in-hospital mortality of adrenal crisis is <1%, timely and adequate treatment for acute AI seems to be successful in most cases, highlighting that a delay in diagnosis is the main determinant of mortality (18).

The incidence of adrenal crisis varies between 4.1/100PY to 9.3/100PY in well-conducted studies among AI patients on standard glucocorticoid replacement therapy (15, 19–21), although there is large variety in interpatient susceptibility (21, 22). These differences in interindividual susceptibility are not well understood (23). Well-described factors that increase the risk of adrenal crisis are a history of a previous crisis (15, 22), as well as higher age, hypogonadism, and diabetes insipidus, the latter by increasing dehydration and as reflection of more extensive pituitary damage (5, 24, 25). Although glucocorticoid receptor polymorphisms that affect glucocorticoid sensitivity potentially alter the susceptibility to adrenal crisis, only one study investigated this before (*i.e.* Bc/l polymorphism), and did not detect a clear association (26). Several subgroups of patients with different AI etiology are also known to be more vulnerable to adrenal crises, which we will further elaborate in *Specific Subgroups With Higher Vulnerability for Adrenal Crisis*.

### Pathophysiology

The physiological effects of acute cortisol deficiency are enormous, and are accompanied by a wide spectrum of signs and symptoms, occurring already after a few hours of cortisol deprivation due to a short half-life of cortisol levels of 90 minutes (23). In line with the known biological effects of cortisol, patients generally develop an adrenal crisis within several hours, and even more rapidly in children (19).

The exact pathophysiological processes that underlie an adrenal crisis are not fully elucidated. First of all, the normal suppressive control of endogenous glucocorticoids on pro-inflammatory cytokines is lost, leading to a strong cytokine release, in combination with an increased sensitivity to the toxic effects of these cytokines. This extensive cytokine release is responsible for the systemic inflammatory response during an adrenal crisis that manifests with fever, anorexia, and diffuse pain (19, 23). These pro-inflammatory cytokines impair glucocorticoid receptor function, further aggravating the cortisol deficiency. Next, the lack of permissive action of glucocorticoids on adrenergic receptors/catecholamine action leads to vasodilatation and severe hypotension (27, 28). In primary AI, this process is reinforced by impaired sodium and fluid retention as a result of concomitant mineralocorticoid deficiency (29, 30). Volume depletion can be further increased by vomiting and diarrhea, altogether explaining the electrolyte disturbances during a crisis. Moreover, the absence of glucocorticoid-induced gluconeogenesis can lead to hypoglycemia, especially in children.

### Definition of Adrenal Crisis

To date, there is still no golden standard to ‘prove’ presence of an adrenal crisis. Moreover, there is no universally accepted definition, and thereby, in current clinical practice, acute clinical impairments in patients with established AI are often marked as adrenal crises (23, 31). The absence of a good definition and the significant overlap with milder episodes of hypoadrenalism, may impede early recognition, and adequate management of an adrenal crisis. This may especially be a problem for health care professionals less experienced in endocrinology. Moreover, the use of various definitions hampers correct comparison of adrenal crisis incidence between studies, and impedes evaluation of the effects of different intervention strategies.

In this respect, we believe that the proposed definition of Allolio and colleagues is very useful in clinical practice (19), defining an adrenal crisis as: a combination of (1) a profound impairment of general health, with (2) at least two of the following conditions: hypotension (systolic blood pressure <100 mmHg), nausea or vomiting, severe fatigue, hyponatremia, hypoglycemia, and hyperkalemia, needing subsequent parenteral glucocorticoid administration (19). Finally, the recovery of symptoms after parental glucocorticoid administration is included in this definition, although the exact time within symptoms have to respond has not been clearly specified.

Another widely used definition is the one of Rushworth and colleagues, stating that an adrenal crisis is an acute deterioration in health status associated with absolute (systolic blood pressure <100mmHg) or relative hypotension (systolic blood pressure ≥20mmHg lower than usual), with features that resolve within one or two hours after parental glucocorticoid administration (23). An advantage of this definition is that also relative hypotension is included, as well as inclusion of a time frame in which symptoms have to respond on steroid administration. On the other hand, clinical symptoms are not well specified, with scope for interpretation of the treating clinician, and electrolyte disturbances are not included in this definition.

The definition used in the recent overview article of Nowotny et al. on behalf of the European Reference Network on Rare Endocrine Conditions (Endo-ERN) is based on a combination of previous definitions including the above mentioned, and additionally includes fever and somnolence in the symptoms, whereas relative hypotension, and recovery after steroid administration are just not included (32).

Based on available literature, we would like to propose a new definition of adrenal crisis with the above mentioned definitions as a starting point, with several refinements, as presented in **Table 1**. We mainly added relative or postural hypotension, while we believe that this is also a clear indicator for volume depletion, and a time frame of 1 hour in which patients have to respond to intravenous glucocorticoid replacement. We also included somnolence in the clinical signs. Ideally, this proposed definition should be studied and validated prospectively in patients with different causes of AI, since probably other definitions (or with a different focus) may fit better for specific subgroups.

**Table 1:** Proposed modified definition and grading system of adrenal crisis.

**Adrenal crisis**
A combination of: (1) a profound impairment of general health, (2) at least two of the following signs/symptoms: hypotension (systolic blood pressure <100 mmHg), relative hypotension (systolic blood pressure ≥20mmHg lower than usual), or postural hypotension; nausea or vomiting, severe fatigue, somnolence, hyponatremia, hypoglycemia, and hyperkalemia, (3) recovery of symptoms after (parental) glucocorticoid administration within two hours
**Suspected/incipient adrenal crisis** (1) subjective symptoms not mentioned above but considered by patient / team as suspected, and (2) recovery after (parenteral) glucocorticoid administration within two hours
**Grading system** Grade 1 Outpatient care only Grade 2 Treatment at emergency department only, without admission to a ward Grade 3 Hospital care on a general ward Grade 4 Admission to an Intensive Care Unit Grade 5 Death as a result of adrenal crisis (with or without glucocorticoid administration)

Based on definitions of Allolio et al. EJE 2015, Rushworth et al. NEJM 2019, and Nowotny et al. Endocrine 2021, and grading system of Allolio et al. EJE 2015.

Although a clear definition may be very helpful in clinical practice, our experience is that patients may have complaints which they link to glucocorticoid deficiency/recognize as an adrenal crisis without completely meeting abovementioned criteria. We advise that in case of suspicion of an adrenal crisis, also when not meeting all criteria, treatment with intravenous glucocorticoids is justified with a low threshold. Then, patients could be classified as having symptomatic AI or an incipient adrenal crisis (23) (**Table 1**).

Next to the necessity of having a clear definition, we propose implementation of a grading system in order to classify adrenal crisis to the type of treatment needed and its outcome, as introduced by Allolio et al. (19), since differences in severity and setting of treatment of the adrenal crisis are associated with concomitant differences in clinical outcomes. The grading system of Allolio et al. makes a distinction between: grade 1, outpatient care only; grade 2, hospital care on a general ward; grade 3, admission to an intensive care unit (ICU); grade 4, death as a result of adrenal crisis (with or without glucocorticoid administration) (19). We propose to add a fifth category between grade 1 and 2 for patients who only need treatment at the emergency department without additional admission to a ward, and thereby modified the grading system of Allolio et al. as represented in **Table 1**.

### Clinical Presentation

#### Symptoms and Signs

Patients with an adrenal crisis often present with gastrointestinal symptoms, including anorexia, nausea, vomiting and (severe) abdominal pain, sometimes leading to an incorrect diagnosis of gastro-enteritis or even an acute peritonitis resulting in unnecessary surgical interventions (19, 33, 34). The underlying pathophysiology of the gastrointestinal symptoms in AI is largely unknown. Part of the explanation could be the noradrenergic overstimulation, since the normal stress response is based on two pillars: the HPA-axis and the autonomic nervous system, of which only the first is deficient in AI, whereas the latter is intact (see *Challenges in Current Treatment Strategies*) (35). Chronic gastrointestinal symptoms are more prevalent in AI patients compared to controls, being incapacitating and similar to symptoms of the irritable bowel syndrome in about 30% of patients (36). Diffuse limb and back pain are also frequently reported. In addition, fever is a common symptom as a result of a concomitant infection or direct consequence of the severe cytokine release during a crisis. However, this systemic inflammation response can be masked by a severe decrease in circulating volume, and, thereby, even result in hypothermia. Patients often experience severe fatigue, lack of energy and dizziness, all being symptoms that can also be observed during milder states of hypoadrenalism. Neurocognitive symptoms can range from concentration problems, agitation and depressive feelings to an overt delirium or even coma in advanced stages, mimicking the neurocognitive symptoms as we know from hypoglycemic states. These neurocognitive symptoms, being a direct consequence of glucocorticoid deficiency in the brain (35), significantly hamper adequate stress management. Another important symptom is profound (postural) hypotension, which can progress to severe hypovolemia and shock. In addition, a small subset of patients develops a reversible cardiomyopathy, having a similar underlying mechanism to a Takotsubo cardiomyopathy. This is a direct result of the glucocorticoid deficiency, normally protecting the heart to noradrenergic effects, the heart is exposed to too high catecholamine levels (37). Clinical deterioration may progress quickly: generally within several hours.

In case of undiagnosed AI, symptoms can be easily masked until a patient presents with an overt adrenal crisis triggered by a stressful event (1). There is generally a gradual deterioration of general well-being over a period of weeks to months, or sometimes even years, with progressive postural hypotension, fatigue, weight loss, and anorexia. These complaints may be accompanied by multiple doctor’s consultations with extensive clinical investigations, often resulting in misdiagnosis, in particular with psychiatric illnesses such as anorexia nervosa or a depressive disorder (33). Characteristic signs of primary AI are salt craving and the development of distinctive diffuse hyperpigmentation of the skin, predominantly on friction points, sun exposed areas and mucous membranes. The hyperpigmentation is the consequence of proopiomelanocortin (POMC) overproduction, being the precursor of both adrenocorticotropic hormone (ACTH) and melanocyte-stimulating hormone (MSH) (29).

#### Biochemical Abnormalities

During an adrenal crisis, standard laboratory tests may show various electrolyte disturbances, including hyponatremia, hyperkaliemia and hypercalcemia, but a pitfall is lack of abnormality in some cases. In primary AI, hyponatremia is mainly the result of concomitant aldosterone deficiency, leading to natriuresis and volume depletion, and also hyperkaliemia. In secondary AI, hyponatremia is due to failure of vasopressin suppression and impaired electrolyte-free water excretion in the kidneys (29). The hypercalcemia is caused by reduced calcium excretion and increased bone resorption during an adrenal crisis (29), and can be aggravated by volume depletion. In addition, variable degrees of a renal insufficiency can be observed as a result of hypovolemia, and hypoglycemia can be present due to reduced gluconeogenesis. Other laboratory abnormalities include a mild normocytic anemia, increased inflammatory markers and altered immune-cell populations, such as neutropenia, lymphocytosis or eosinophilia (23).

#### Precipitating Factors

**Figure 1:**
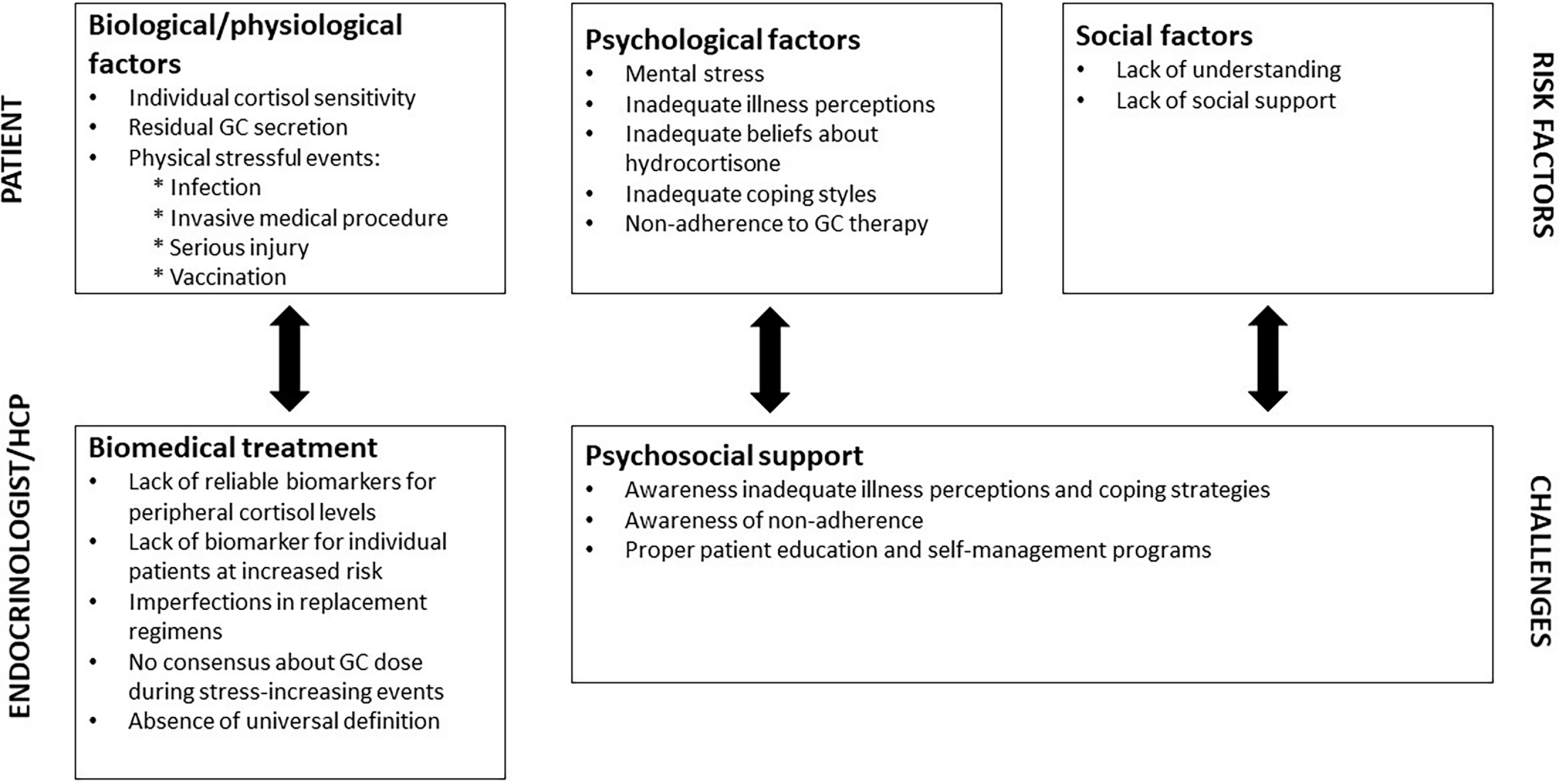
Biopsychosocial risk factors and challenges from the perspective of patients and healthcare professionals. HCP, health care professional; GC, glucocorticoids.

Actually all conditions that increase the glucocorticoid demand can trigger an adrenal crisis, in which a distinction can be made between biomedical, psychological and social factors (**Figure 1**). Infections are, by far, the most common cause of hospital admissions and death due to adrenal crisis (14, 15, 18, 20, 22, 38). In older patients, bacterial infections play a main role (*i.e.* gastro-enteritis, bronchopulmonary and urogenital infections), whereas viral infections are common triggers in children (15, 18, 20, 39). Especially gastro-intestinal infections are notorious for severe adrenal crises by compromising the intake and intestinal absorption of oral glucocorticoids, and aggravated hypovolemia due to vomiting and diarrhea. It should be noted that the gastro-intestinal symptoms of an adrenal crisis are also regularly misdiagnosed as gastro-intestinal infection (20, 23).

Other conditions that frequently precipitate adrenal crises are (major) surgery, as well as dental procedures, pregnancy/labor, serious injury or accidents, exhaustive physical activity, vaccinations, extreme weather conditions, and emotional or mental stress (22, 40, 41). In addition, initiation of levothyroxine replacement in case of hypothyroidism in a patient with an undiagnosed AI can trigger an adrenal crisis, since the metabolic demand is accelerated with thyroid hormone replacement, thereby overcharging the cortisol supply (23, 42). Since thyroid dysfunction is a common comorbidity in (primary) AI, it is of great importance that doctors are well informed to initiate glucocorticoid replacement before the start of thyroid hormone replacement. Also a coexisting thyrotoxicosis can precipitate an adrenal crisis (43).

Several medication groups are known to increase the risk for adrenal crisis, including a number of chemotherapy or immunotherapy forms. Mainly immune-checkpoint inhibitors are notorious for their endocrine complications, since these agents can induce both a hypophysitis and adrenalitis with subsequent AI (44). The full spectrum of clinical relevant endocrine disease, including adrenal crisis, in this group needs to be established. Also barbiturates or adrenostatic drugs such as etomidate, mitotane, or ketoconazole can increase the risk of an adrenal crisis (22, 45, 46). Cytochrome P450 3A4 inducers (*i.e.* mitotane, carbamazepine, rifampicin) increase the metabolism of hydrocortisone, thereby increasing the hydrocortisone demand (47). On the other hand, hydrocortisone dose adjustments are also indicated when prescribing CYP450 3A4 inhibitors (*i.e.* ritonavir, grapefruit juice) to AI patients to prevent over replacement (19).

An important patient-related factor related to increased vulnerability to adrenal crisis is non-adherence to glucocorticoid replacement therapy, which has been previously reported in any form in up to 85% of patients (48) (see *The Patient’s Perspective*). Patients may forget or may be unaware of a stressful event and do not increase doses in case of stress, or tend to underdose themselves because of fear for side effects. In this respect, glucocorticoid deficiency in the brain significantly hampers adequate stress management in these patients in case of adrenal crisis, as mentioned before. Clinician-induced sudden cessation or too rapid tapering of chronic steroid therapy can also trigger adrenal crisis (49, 50). In about 10% of the adrenal crises, no clear trigger can be identified despite thorough evaluation (15, 19).

### Specific Subgroups With Higher Vulnerability for Adrenal Crisis

There are several subgroups of patients with AI that are specifically vulnerable to the development of adrenal crisis. We will discuss several of these subgroups separately, including potential underlying mechanisms that may explain this higher susceptibility, as well as specific points of attention in the management of these subgroups (**Table 2**).

**Table 2:** Specific subgroups of AI patients with higher vulnerability to adrenal crisis.

Subgroup	Incidence adrenal crisis	Specific points for attention
Primary AI	4.7 – 7.6/100PY	Mineralocorticoid deficiency
		Concomitant endocrinopathies
Primary AI and concomitant endocrinopathies*		
*T1DM*	12.5/100PY	Glycemic disturbances
		More bacterial infections
*Hashimoto’s disease*	Unknown	Initiation of levothyroxine: trigger
Cushing’s syndrome	4.1 – 9.3/100PY	Withdrawal syndrome, high early postoperative incidence of adrenal crisis
*After BLAx*		Complete lack of glucocorticoids and mineralocorticoids after BLAx
Adrenocortical carcinoma and mitotane use	Unknown	Severe hypovolemia due to concomitant chemotherapy with vomiting/diarrhea
The elderly patient with multimorbidity	4.4/100PY (NL)	Atypical presentation
	24.3 – 35.3 – 45.8/10^6 in resp. 60-69yr, 70-79yr, 80+yr (Australia)	Higher mortality
		Complicated management, polypharmacy
Pregnancy	9 of 128 pregnancies (7%)	Unreliability of biochemical markers
		Hyperemesis gravidarum and' labor: trigger

*These endocrinopathies could be in the context of PST2

AI, adrenal insufficiency; T1DM, type 1 diabetes mellitus; PST2, polyglandular syndrome type 2; BLAx, bilateral adrenalectomy; PY, person-years

#### Primary Adrenal Insufficiency

A number of studies, mostly of retrospective design, report that primary AI patients are at increased risk of adrenal crisis when compared to secondary AI. The adrenal crisis incidence in primary AI ranges from 4.7/100PY to 7.6/100PY between different studies (20–22, 51–53) (15), although one study reported comparable incidence rates (54).

This increased vulnerability to adrenal crisis can be explained by several factors. First of all, most primary AI patients require both glucocorticoid and mineralocorticoid replacement, resulting in more pronounced circulatory failure during a crisis than in case of glucocorticoid deficiency only. In this respect, it should be mentioned that in high doses, several glucocorticoids including hydrocortisone can exert mineralocorticoid effects as well, whereas in doses within the physiological range 11β-hydroxysteroid dehydrogenase type 2 oxidizes cortisol rapidly to the inactive metabolite cortisone in mineralocorticoid target tissues (see *Management of Adrenal Crisis*). Moreover, whereas most primary AI patients completely lack glucocorticoids, a significant subset of secondary AI patients have residual cortisol secretion as expression of partial HPA-axis insufficiency only. Finally, a substantial proportion of primary AI patients have concomitant autoimmune diseases, being an independent risk factor for adrenal crisis (*see below*).

#### Primary Adrenal Insufficiency With Concomitant Autoimmune Endocrinopathies

Several reports revealed that the combination of type 1 diabetes mellitus (T1DM) and primary AI also increases vulnerability to adrenal crisis compared to primary AI alone (52, 53, 55). An Australian study reported an adrenal crisis incidence of 12.5/100PY in diabetic AI patients *vs* 4.7/100PY in non-diabetic AI patients (53), which is quite consistent with another report from a German University Hospital showing exactly the same adrenal crisis incidence of 12.5/100PY in AI patients with concomitant T1DM (52).

While the exact underlying mechanisms for this higher vulnerability are unknown, it is hypothesized that behavioral factors play a role, since managing two disorders simultaneously during acute illness is a challenge, and glucocorticoid deficiency in the brain hampers adequate stress management (35), as well as opposing effects of insulin on glucose metabolism (53). Misalignment between insulin and glucocorticoid replacement may result not only in adrenal crisis, but also in hypo- or hyperglycemia, and diabetic ketoacidosis. Prevalence of infections is also higher in patients with combined AI and T1DM leading to more frequent hospital admissions (55). HR-QoL has been reported to be significantly lower in patients with both AI and T1DM compared to AI alone (56), and the constant fear of adrenal crisis is likely to be a contributing factor.

In line with the reports presented above, patients with polyglandular syndrome type 2 (PST2) are specifically vulnerable to adrenal crises, when compared to primary or secondary AI alone, with an incidence of respectively 10.9/100PY *vs* 7.6/100PY *vs* 3.2/100PY (52). Although specific endocrine expressions of PST2 have not been clearly differentiated in this report, in our experience, not only the combination of T1DM and AI increases vulnerability to adrenal crisis, but also the combination of Hashimoto’s disease and AI. In current literature, the incidence of adrenal crisis in patients with combined Hashimoto’s disease and AI has not been well studied and consists mainly of anecdotal evidence of case reports. A facilitating factor to the increased vulnerability could be the relative overreplacement with levothyroxine in case of increased glucocorticoid need, being a well-known trigger of adrenal crisis (23, 42, 43).

In addition, primary AI (whether or not in the context of a polyglandular syndrome) is also associated with non-endocrine autoimmune diseases. In this respect, in patients with combined AI and celiac disease, ulcerative colitis or Crohn’s disease the gastrointestinal absorption of hydrocortisone is compromised, increasing the vulnerability to adrenal crisis (57–59), especially during a flare, which is thereby a clear trigger for an adrenal crisis.

#### After Treatment for Cushing’s Syndrome Including Bilateral Adrenalectomy

The incidence of adrenal crisis after surgical treatment for Cushing’s syndrome ranges from 4.1 to 9.3/100PY between different studies, and most reports, including the meta-analysis of Ritzel and colleagues, show that these patients have an increased risk of adrenal crisis (24, 60–62). Vulnerability is highest in the subset of patients with a history of bilateral adrenalectomy for Cushing’s syndrome, since these patients completely lack both endogenous glucocorticoid and mineralocorticoid secretion (62). The risk of adrenal crisis is also increased in patients with a history of bilateral adrenalectomy for other indications, for example in case of bilateral pheochromocytoma in the context of multiple endocrine neoplasia 2 (MEN2) syndrome. In patients receiving other forms of treatment for Cushing’s syndrome with different etiology, for example transsphenoidal surgery or radiotherapy for ACTH-dependent Cushing’s syndrome, the risk of adrenal crisis mainly depends on the individual’s residual glucocorticoid secretion.

A recent study combining AI patients after surgical treatment for both ACTH-dependent and ATCH-independent Cushing’s syndrome, showed that most adrenal crises occurred within the first months after successful surgery (24). This early postoperative period is believed to be the phase with most severe suppression of endogenous glucocorticoid secretion (24), *i.e*. low basal values and low tendency to peak in dynamic tests in a system still used to elevated cortisol levels. Most patients already receive higher hydrocortisone doses to counteract withdrawal symptoms, while the pulsatile and circadian activity of the HPA-axis/endogenous cortisol secretion recovers. In this respect, it remains a challenge to distinguish postoperatively between symptoms of hypoadrenalism and symptoms that are the consequence of glucocorticoid withdrawal or persistent symptoms despite reversal of cortisol excess, since symptoms may be similar (63). In this respect, it has been shown previously that (partial) recovery of the HPA-axis activity can also occur years after surgical reversal of cortisol excess (64, 65).

#### Patients Using Mitotane for Metastatic Adrenocortical Carcinoma

Patients treated with mitotane in the adjuvant setting of a metastatic adrenocortical carcinoma are another highly vulnerable group with respect to development of adrenal crisis. Although the exact incidence of adrenal crisis in this setting is unknown, several case reports describe a fulminant adrenal crisis in mitotane-treated patients that demonstrate a hormonal tumor response (66, 67).

Mitotane, or dichlorodiphenyldichloroethane, is not only an adrenostatic drug leading to adrenal necrosis and thereby AI, but is also known to induce the cytochrome P450 enzyme 3A4, thereby increasing the metabolism of hydrocortisone. Therefore, during treatment with mitotane, it is necessary to increase the hydrocortisone dose to unusually high levels to prevent under replacement (47, 67). Sometimes, patients also require mineralocorticoid replacement due to enhanced destruction. During use of mitotane, free cortisol and ACTH should be determined instead of total cortisol levels, since mitotane increases plasma levels of steroid binding proteins. Another complicating factor is that next to mitotane, which itself frequently leads to gastro-intestinal side effects, most patients receive concomitant adjuvant chemotherapy, leading to mucositis with profuse vomiting and diarrhea that further increases hypovolemia and the inability to take oral hydrocortisone. Therefore, despite preventive higher hydrocortisone doses, patients still remain at increased risk of adrenal crisis.

#### The Older Multimorbid Patient With Adrenal Insufficiency

We believe that the elderly AI patient with multimorbidity also receives specific attention, since mortality attributable to adrenal crisis increases significantly with higher age (39, 68). Although only few studies examined adrenal crisis rates in the elderly, a population-based study revealed that AI patients ≥60 years have the highest age-specific incidence of adrenal crisis, showing an increasing incidence with increasing age, whereas another study did find a comparable incidence between patients <65 and >65 years in a multivariable model correcting for several comorbidities (4.4/100PY) (20, 25).

Several factors might explain an increased vulnerability to adrenal crisis in older patients. First, prevalence of comorbidities is higher, mainly the frequency of (severe) bacterial infections. Moreover, older patients have a reduced tendency to develop fever and also other symptoms can be mitigated, contributing to the diagnostic delay (18, 39). In addition, AI symptoms may be incorrectly attributed to other common diseases, or may be classified as side effect of medication or the consequence of ageing itself. Next to the atypical clinical presentation, cognitive decline, social isolation, impaired vision and hearing, and polypharmacy might complicate management in older AI patients, and may interfere with their treatment compliance (18, 25). In line with this, it has to be mentioned that these factors do not only play a role for older patients, but also for younger AI patients with a poor support system or AI patients with psychiatric illnesses and poor self-care.

#### Tertiary Adrenal Insufficiency

A main problem is the lack of awareness of tertiary or glucocorticoid-induced AI by clinicians in patients with long-lasting exogenous steroid use (69, 70). Therefore, this subgroup is, especially in the untreated phase, at specific risk of adrenal crisis during stressful situations, despite a residual endogenous cortisol production in many patients with the ability to (partially) recover within a variable period after cessation of steroids. However, literature on the incidence of adrenal crisis in tertiary AI is scarce. Moreover, available studies are very heterogenous with respect to etiology of tertiary AI (*i.e.* steroids for rheumatologic diseases, high-dose steroids lymphoblastic leukemia or inhalation steroids for asthma) (71, 72), and criteria for AI and adrenal crisis, and report inconsistent results (20, 51). Interestingly, one study of Smans et al. reports a remarkable high incidence of adrenal crisis among patients with glucocorticoid-induced AI, namely 15.1/100PY (20), although the number of patients with tertiary AI was very small in this study (only 28 patients). In the study of Li et al. the risk of adrenal crisis in tertiary AI was comparable with the risk observed in secondary AI patients (51). Further studies are needed to investigate the risk exact of adrenal crisis in the different subgroups of patients with glucocorticoid-induced AI. The focus should be on identification of individual patients with long-term exogenous steroid exposure that are most at risk for adrenal crisis in order to give adequate individualized stress instructions (70).

#### Pregnant Patients With Adrenal Insufficiency

Pregnant patients with AI are also increasingly vulnerable to adrenal crisis. First, diagnosing new onset AI during pregnancy is complicated by symptoms that are falsely attributed to the pregnancy itself as well as unreliability of several biochemical parameters during pregnancy. Pregnancy can be considered as a state of hypercortisolism due to general increase in the activity of the HPA-axis, and is associated with a gradual but significant increase of corticosteroid-binding globulin and total serum cortisol concentrations. During the third trimester, free cortisol concentrations rise, altogether explaining the need for higher hydrocortisone doses in AI patients (2.5 to 10mg increase of daily dose, 20 to 40% between different studies) (1, 73). Since serum progesterone has anti-mineralocorticoid effects, fludrocortisone dose often needs also to be increased in the third trimester in patients with primary AI (74). Although literature on the incidence of adrenal crisis among pregnant AI patients is scarce, a recent multicenter study of Bothou et al. described the course of 128 pregnancies among 113 women with different AI etiology (73). In this report, glucocorticoid dose was increased in 57% of patients during pregnancy, whereas mineralocorticoid dose was increased only in a minority of patients. Moreover, adrenal crisis was reported in 9 out of 128 patients (7%) during pregnancy, which was triggered by hyperemesis gravidarum in a significant proportion of patients (73).

The endocrinologist has an important role in providing the patient and its obstetric team with a written therapeutic plan on the intravenous glucocorticoid stress scheme during pregnancy but especially during delivery, since the latter is a well-known trigger for adrenal crisis. Although there is no evidence on the exact timing and dosage of glucocorticoid coverage peri- and postpartum with room for interindividual adjustments, it is recommended to start with an initial bolus of 100mg hydrocortisone intravenously before the active phase of labor, followed by continuous infusion or repeated boluses every 6 hours (73, 75).

### Management of Adrenal Crisis

The cornerstone in the treatment of a suspected adrenal crisis is immediate parental administration of glucocorticoids and prompt rehydration. An initial bolus of 100mg hydrocortisone is recommended, preferably intravenous, otherwise intramuscular, without further delay for waiting for laboratory results or biochemical proof in patients with previously undiagnosed AI (19, 76). When intravenous hydrocortisone is not available, administration of prednisolone or other synthetic glucocorticoids are reasonable alternatives. Next to glucocorticoid administration, adequate fluid resuscitation with isotonic saline is necessary, with the advice to administer 1000ml of 0.9% sodium chloride during the first hour. Additional fluid administration should be based on the individual patient’s need, paying attention to avoidance of fluid overload and electrolyte disturbances. The combination of intravenous steroids and isotonic saline results in an almost invariably quick improvement of signs and symptoms (19, 32). If patients do not adequately respond within the first 24 hours after glucocorticoid administration, an alternative diagnosis, such as sepsis or a cardiogenic event, or coexisting cause of hypotension should be considered (23).

Next, it is essential to identify and treat the underlying cause for the adrenal crisis. Further thorough investigation should focus on identifying an underlying infection, since this is by far the most common precipitating factor for an adrenal crisis and a substantial cause of death. It is essential to take cultures adequately and start antibiotic treatment in case of a suspected bacterial infection.

Based on the severity of the adrenal crisis, its response to initial treatment, and the intercurrent illness, a distinction must be made between patients that can be managed safely in an outpatient setting with closely follow-up by a specialized nurse and patients that need in-hospital care on a regular ward or even the Intensive Care Unit (see grading system, *Definition of Adrenal Crisis*). Currently, there are no available prognostic factors or biomarkers that can reliably predict whether patients need in-hospital or out-hospital care, so this is mainly based on the clinical judgement of the physician. When in-hospital treatment is justified, a continuous hydrocortisone infusion of 100 to 200mg/24 hours can be given, after the initial bolus as described above. Although there is no evidence for the exact steroid dose required in the acute treatment of adrenal crisis, is has been shown that continuous intravenous hydrocortisone was the only delivery mode that steadily maintained circulating cortisol levels within the physiological range observed during major stress, whereas intermittent administration of hydrocortisone boluses resulted in frequent drops with lower cortisol concentrations, thereby having the potential risk of (periods of) under replacement (77) (see *Challenges in Current Treatment Strategies*). This continuous infusion can be tapered gradually based on the clinical condition of the patient. After hospital discharge, or when patients are primarily treated in an outpatient setting, the daily oral glucocorticoid dose is generally doubled for a few days until the trigger of the adrenal crisis has been resolved; then, patients return to their standard dosage schemes (32).

In case of primary AI, additional mineralocorticoid supplementation should be initiated in the form of fludrocortisone when hydrocortisone doses are tapered below 50mg a day. During the initial treatment with high hydrocortisone doses, no additional fludrocortisone is needed due to sufficient action at the mineralocorticoid receptor of supraphysiological hydrocortisone dosages (76).

## Challenges in Current Treatment Strategies

Despite new developments in the treatment of AI patients during the last years, with more individualized hydrocortisone replacement strategies, the introduction of long-acting hydrocortisone preparations, and increasing awareness for adequate prevention, both the endocrinologist and the patient still face many challenges in every day’s management (5, 19, 32, 51, 78). Here, we will illustrate several important challenges of the doctor regarding the current care of AI patients, and in the next paragraph we highlight unmet needs from the patient’s perspective.

### Lack of Reliable Biomarkers Reflecting Peripheral Cortisol Concentrations

As previously noted, the optimization of individualized glucocorticoid dosing warrants development of reliable biomarkers truly reflecting tissue cortisol concentrations (5). A promising, well-studied biomarker to reflect peripheral cortisol exposure over longer periods is hair cortisol. This tool has previously been shown to be a reliable reflection of tissue cortisol exposure in various patient groups, including AI patients on chronic glucocorticoid replacement (12, 79–81). Previous studies showed that both primary and secondary AI patients on chronic hydrocortisone replacement had significantly higher hair cortisol levels compared to pituitary patients without AI and healthy controls (81). This underlines the non-physiological nature of current replacement regimes with mild over replacement in a large number of patients. Although this biomarker has been showed to adequately reflect peripheral cortisol concentrations, it has not yet been integrated into clinical practice.

Other potential suitable biomarkers in this respect are cortisol and cortisone measurements in saliva and subcutaneous fat, although especially the results on the reliability of salivary cortisol and cortisone area-under-the curve (AUC) levels are contradictory between studies (82–84). Another recent study investigated changes in gene expression following different glucocorticoid replacement doses as a novel biomarker to guide glucocorticoid replacement, showing promising results with several candidate transcriptional biomarkers (85). Further research should aim on the validation and development of practical biomarkers to assist in improved individual replacement regimes.

### Intrinsic Imperfections of Current Glucocorticoid Replacement Regimes

Over the last few decades, there is increasing awareness for the observed lower HR-QoL and premature death in AI patients as a direct result of intrinsic imperfections in hydrocortisone replacement (5, 9). Since current replacement strategies cannot mimic physiological hormonal secretion patterns, and, since many factors are known to affect individual glucocorticoid sensitivity (*i.e.* other medications, glucocorticoid receptor polymorphisms), it remains difficult to establish an optimal replacement dose for the individual patient (10, 11). This is further complicated by the lack of biomarkers that accurately reflect tissue cortisol concentrations, resulting in a substantial proportion of patients being chronically under- and overtreated with significant morbidity in both scenarios (12). Insufficient cortisol coverage results in chronic noradrenergic activation with a lot of complaints and the risk of a potentially fatal adrenal crisis during increased stress, as intensively described above, whereas even subtle glucocorticoid overexposure results in a Cushing-like phenotype with higher hydrocortisone levels being associated with impaired QoL (12). In agreement with this finding, the adverse metabolic, cardiovascular, and psychological effects of long-term supraphysiological glucocorticoid replacement need specific attention, since there are significant associations between chronic over replacement and diabetes mellitus type 2, obesity, osteoporosis, psychological morbidity, maladaptive personality traits, and premature cardiovascular death, respectively (5, 7, 86, 87, 70).

To better mimic the normal circadian cortisol rhythm in AI patients on chronic hydrocortisone replacement, several new, promising, slow-release glucocorticoid preparations have been developed, such as Plenadren, a dual-release system, and Chronocort, being a modified-release preparation (32). These new, long-acting glucocorticoid agents are able to better mimic physiological diurnal cortisol levels with exception for the rise in cortisol levels prior to awakening (88–90), and reduce overall cortisol exposure with less negative effects on anthropometric parameters (91, 92). Chronocort is prescribed twice a day and is able to perfectly mimic physiological cortisol levels during the day, including the morning cortisol peak (93). Another option that better mimics the pulsatile secretory pattern is a subcutaneous infusion system, although these pumps are currently not used in clinical practice (94).

### No Consensus on Optimal Glucocorticoid Dose During Different Stress-Increasing Events

There is no dispute about the necessity of AI patients to increase their hydrocortisone dose adequately during episodes of (major) stress, but there is no consensus on the optimal glucocorticoid dose during different stressful events, and its delivery mode. Therefore, recommendations on glucocorticoid stress doses are mostly empirical than evidence-based, thereby being quite variable, and are based on expected stress in specific situations (31, 95). The physiological response of the HPA-axis during stress has been most extensively investigated in patients undergoing surgery, whereas in other forms of stress, such as infections or emotional stress, the normal physiological response is less clear and more variable (96) (77). Despite the scarcity of well-designed studies on the exact physiological cortisol response in different stressful situations, the recently published overview article of Nowotny et al. on behalf of the Endo-ERN, which includes representatives from patient associations, provides a very useful and practical overview explaining the sick day rules and recommendations on the glucocorticoid stress dose to prevent progression to an overt crisis (32). For easy accessibility, the Endo-ERN consensus on guidelines on taking glucocorticoids to prevent adrenal crises is available using practical examples in daily life for both mild, moderate, and severe physical and mental stress in many languages (https://endo-ern.eu/wp-content/uploads/2019/03/20190312-Stressinstructie-addisoncrisis-hydrocortison-ENG-Endo-ERN-approved.pdf).

### Absence of Universally Accepted Definition of Adrenal Crisis

As we already have discussed in *Definition of Adrenal Crisis*, we emphasize the necessity of a clear definition of an adrenal crisis to facilitate prompt recognition of this clinical condition as well as proper comparison of incidence rates and evaluation of care between (subgroups of) patients.

### Lack of Tools to Identify Individuals at Increased Risk of Adrenal Crisis

Since there are currently no reliable laboratory tests or other (bio)markers to adequately predict an adrenal crisis, it remains a challenge for the endocrinologist to identify individual patients with increased vulnerability. Therefore, clinical tools are needed to detect individuals at increased risk and, ideally, also to detect prodromal periods of an adrenal crisis, enabling the clinician to timely interfere and prevent progression into an overt crisis.

A study of Meyer and colleagues investigated the role of longitudinal disease-specific QoL questionnaires (*i.e.* AddiQoL) in detecting prodromal periods of an adrenal crisis (97). This study shows that score deviations in longitudinal AddiQoL assessments may help patients in detecting their risk of an adrenal crisis, and thereby in the identification of patients at increased risk of adrenal crisis. In this respect, improving the patient’s awareness of early symptoms of AI or an imminent crisis by regular self-evaluation of HR-QoL *via* apps or a diary may help patients to adequately adjust the hydrocortisone dose and timely contact their endocrinologists for further instructions. Other clinical tools to detect predict an adrenal crisis or detects its prodromal symptoms, are currently unavailable.

### No Evidence on Effectivity of Current Patient Education Strategies

The consultant endocrinologist and/or the specialized nurse is the first person to inform and educate patients on how to recognize an adrenal crisis, its potential risks, and training on how to handle in case of stressful events. In addition, patients can be referred to written information or online information available *via* patient societies (*Bijniernet*, https://www.bijniernet.nl/medicatie-themapagina/stressinstructies/). This first education is considered to be essential to improve both patients’ adherence and skills, and is considered as one of the cornerstones in the prevention of an adrenal crisis (32, 76). However, literature about the effectivity of specific patient education modalities in the prevention of adrenal crisis remains scarce, and the most appropriate method to effectively educate patients remains subject to debate.

A previous study showed that one or two educational consults did not improve self-management skills to prevent adrenal crisis (98). Patients reported that insufficient handling in stressful situations was mainly the consequence of lack of experience of stress-increasing circumstances and not remembering how to act/not be able to act due to severe weakness and cognitive dysfunction in the context of the adrenal crisis (21, 22, 51, 98, 99). In this respect, it is important to emphasize the particular vulnerability of patients with AI in case of development of adrenal crises: glucocorticoid deficiency in the brain can also hamper adequate stress management (instructed dose increase or parenteral administration), like this is the case in patients with diabetes and hypoglycemia (35). A remarkable finding was that 60% of patients felt themselves to be well informed on how to act during a stressful situation, but this did not correlate with the ability to act appropriately during a stressful situation, especially in older patients, likely be a consequence of glucocorticoid deficiency in the brain (99, 100). Therefore, an individualized approach is needed for the (extent and dosing of) stress instructions (100), and it is strongly advised to involve family members in the training sessions and to supplement oral instructions with written information. Other reports have shown that repeated group education might be a more effective tool to improve self-management skills and proper use of stress-related glucocorticoid dose adjustments in AI patients (101, 102). Ninety-five percent of patients reported an improved subjective well-being after participation in a patient group education program, also 6 to 9 months after completion of the program (101).

Self-care of patients and adequate glucocorticoid dose adjustments are the key to success in the prevention of adrenal crisis. In this respect, steroid emergency cards are very important to emergency clinicians, but currently underused (103). These cards have already been successfully harmonized within Europe and are available in different languages (32, 92). In addition, only a small proportion of patients is equipped with an emergency set, and also in patients traveling with an emergency kit, barriers to self-injection are great (103).

Another important aspect that merits attention is the non-adherence to treatment, being a problem in up to 85% of AI patients. Non-adherence has been most frequently reported among patients who expressed dissatisfaction with information on glucocorticoids and concerns about glucocorticoid side effects (48). It is important for endocrinologists to identify such inadequate beliefs about medicines, since this could help the doctor to engage patients in treatment decisions and support their adherence (104), and thereby lowering the risk of adrenal crisis.

## The Patient’s Perspective

As previously noted, patients with AI suffer from persistent complaints and need lifelong hydrocortisone replacement therapy. Consultation visits with their endocrinologist are usually scheduled two to three time a year and when needed more visits are planned, but eventually patients and their parents or partners need to manage their disease and the glucocorticoid replacement therapy on their own. Patients reported that the AI and the glucocorticoid replacement therapy resulted in adaptations in their physical activity and their social-, work- and family life. Moreover, 76% of the patients reported concerns about the long-term side effects of their replacement therapy (105). Tiemensma et al. reported that stronger beliefs about the necessity of glucocorticoid replacement therapy were associated with feelings of less personal control over the illness. More concerns about potential negative side-effects of glucocorticoid replacement therapy were associated with perceiving a lower extent to which AI can be controlled (106).

In 2016, Chapman et al. performed a survey study in 81 patients with AI on glucocorticoid replacement therapy assessing how patients perceive treatment and their self-reported adherence to treatment (48). Patients reported dissatisfaction with the amount of information they received about potential negative side-effects compared to the amount of information they receive about the action and use of glucocorticoids. Most of the patients (61.7%) reported to be dissatisfied with the amount of information they received about the risks of getting side-effects. Concerns about the potential side-effect and dissatisfaction with information about the treatment was associated with nonadherence to therapy. Thirty-seven percent of patients reported to take the dose later in the day than advised, 35 percent of the patients reported to take the dose at a different time of the day than advised, and 28 percent of the patients reported to forget taking the glucocorticoids (48). This exemplifies the importance of how patients perceive their illness and their beliefs about glucocorticoid replacement therapy in their self-management skills and adherence to treatment. Moreover, it accentuates the central role of patient themselves in the management and prevention of adrenal crisis.

In addition, a large proportion of patients reports to receive suboptimal care at the emergency department, and many patients have repeated negative experiences, in which an inexperienced care giver plays an important role in this negative experience (51). A large subset of patients feels not well understood by the health care professional, leading to a delay in intravenous glucocorticoid administration (51, 103). A better definition of adrenal crisis would be very beneficial to guide also more inexperienced doctors in making an early and correct diagnosis, and initiate prompt glucocorticoid treatment, which can add to improve the patient’s experience at the emergency room (see *Challenges in Current Treatment Strategies*).

### Focus Group Data in Patients With Adrenal Insufficiency

In 2014, in two focus group sessions of patients treated at our center, a group of six patients with AI participated (3 females, 3 males; age ranged from 32 to 60 years of age). Four patients were diagnosed with M. Addison, whereas AI was diagnosed in one patient after unilateral adrenalectomy for Cushing’s syndrome and in one patient after bilateral adrenalectomy for pheochromocytoma. Duration of follow-up ranged from 1 year to 29 years. The aim of these group conversations was to define the patients’ perspective on QoL. This study was part of a previous focus group study about QoL in patients with pituitary disease [see Andela *et al.* (107)]. Considering the aim of the present review, only categories related to medication adjustments or adrenal crisis will be discussed. The following five categories were derived from the focus group conversations with patients with AI.

Patients reported issues related to adaptations in hydrocortisone intake, such as how and when to increase hydrocortisone intake in certain situations (e.g., going to the dentist, having surgery). Furthermore, they reported feeling sick and more easily irritated in situations when they suddenly require elevated hydrocortisone intake (e.g., having a cold, nausea, a tense feeling).

“*If I don’t feel well, I think better avoid a crisis and double my dose. However, if you have to go to the dentist or dental surgeon, there is no one who can tell you what your medication should be.*”

“*My aunt has diabetes, which is a lot easier. You can measure your blood sugar in order to determine whether the blood sugar in adequate, we have to feel it a lot more. One can also feel hypoglycemia coming, but at least you can measure it.*”

The psychological impact of the risk of having an adrenal crisis: patients did not report a fear of having an adrenal crisis, but they were aware of the risk and take precautions. Patients reported feeling vulnerable and dependent of others during a crisis, and questioning themselves afterwards what they could have done to prevent the crisis. Patients reported that when they are in a crisis they are not able to think and handle properly and experience a degree of apathy.

“*It is not that I am afraid, but that I think logically about what is my risk and what should I take with me. I’m not afraid of it, because otherwise you can’t do anything anymore.*”

“*The adrenal crisis happens to you, when I look back I question myself when should I have had raised the alarm.*”

“*If I really become aware of it, if I feel it, then it is actually already too far, then you are already in a crisis. If I check myself very carefully and put my attention on little things, then I might be able to recognize it.*”

People reported to take into account the risk of an adrenal crisis by several activities in their daily living (coping with the risk of an adrenal crisis). Taking their emergency inject during traveling, telling the dentist that there is an emergency injection in their bag, making a protocol of what to do during a crisis and placing this in a central place in their house, and wearing a tag. Furthermore, patients tell new people or colleagues about their condition out of precaution, so that these people know what to do in case of a crisis. When patients experience an upcoming crisis they always contact someone, such as their partner of the neighbor.

“*I have drawn up a kind of protocol for myself of an A4 that I have on the inside of my kitchen cupboard so that you can see what to do. For example, never leave the house without a thermometer, if I get above 38.5 then I have to take more hydrocortisone and above 39.5 you should not be home anymore.*”

“*When I go on a business trip with a colleague, I will tell him if I don’t get up in the morning, then knock on my door to see how things are going. And if I’m late for work, always give me a call.*”

When patients are having an adrenal crisis and are admitted to a hospital to receive treatment, they reported that after the hydrocortisone bolus they immediately feel better, but also that they have to recover from the heavy treatment afterwards (experiences with the treatment for an adrenal crisis).

“*It is a matter of going to the hospital in the evening, then you get the ‘miracle cure’ because then they will administer an infusion with cortisol and that takes 2 hours. Then you go to sleep and the next morning you say I want to go home.*”

“*I’m really upset after that. At least four days in which I really don’t feel well that I get such a hit from the cortisol. It is a miracle cure and I feel really fine, but after a day, it really bothered me. Last time I just didn’t go to work, I just couldn’t function.*”

Furthermore, patients reported some unmet needs regarding care. Patient would have had better information about what to do in an upcoming crisis and more specifically how to appley an emergency injection.

“*Learning how to inject is also important for my partner, such as what exactly happens during a crisis and what exactly does a crisis entail.*”

“*You feel bad and you feel a crisis coming, what do you do? Do you inject yourself or do you call the hospital and get injected by a doctor. Those kinds of simple instructions. Also simple instructions as may you double your medication.*”

Furthermore, patients reported the experience that some medical doctors (e.g., general practitioners) are not sufficiently educated about AI and what needs to be done in an adrenal crisis. Patients reported the need to speak up for themselves when they were admitted a hospital in case of a potential adrenal crisis.

“*It is very difficult to discover doctors who have any knowledge of the disease outside of the very small group that has seen a patient. You actually have to constantly think along for yourself.*”

“*I’m quite willing to become a super doctor for my own illness, as long as I don’t have to perform any actions.*”

These quotes illustrate the patient perspective of living with AI and the risk of an adrenal crisis. Regarding the unmet needs regarding care, it should be noted that these focus group conversations were held in 2014, while in the last decade the care for patient with AI seriously has improved with better cooperation between patient societies and healthcare professionals and the development of aids to support the understanding of patients and healthcare professionals. Furthermore, an emergency card was developed that can be shown by patients to healthcare professionals in case of an emergency. Although these developments resulted into better care for patients with AI, it is generally known that there is room for further improvement regarding education of patient and their partners, including psychosocial support as a standard resource (78).

## Future Perspectives

As we highlighted in this overview article, there are many remaining important unmet needs of both patients and doctors in current care for AI patients. We believe that improvement of HR-QoL should be the primary focus of both research and clinical care in the upcoming years. To achieve this, the first step is to diagnose glucocorticoid deficiency and adrenal crisis in an earlier phase, based on clear, and universally used definitions. Second, there is a need to improve hydrocortisone replacement strategies, by more individualized approaches of replacement dosage, and prescription of slow release and modified release hydrocortisone preparations in a subset of patients, or perhaps even pulsatile hydrocortisone release *via* subcutaneous pumps in selected cases refractory to conventional treatment. Third, studies integrating PROMs with other biological outcome variables reflecting the biological effects of cortisol at tissue level are needed, including glucocorticoid receptor responsive gene expression in leukocytes, cortisone-cortisol ratio in saliva or subcutaneous tissue, markers of noradrenergic activation, measurement of central nervous system functioning by the use of functional MRI (108), and long-term phenotyping reflecting near normalized cortisol exposure, including parameters for decreased cardiovascular morbidity, neuropsychological dysfunction, and stress resilience in daily life. In addition, integration of the patient’s perspective in current care is extremely important, and as is well illustrated by the quotes of the AI patients in our unique focus groups, more attention is needed for improvement of self-management and knowledge about the disorder of patients and their immediate circle of caretakers (78).

To achieve these goals, close collaboration between health care professionals and patient associations is needed, as well as integration of care, education, and research in reference centers with specific expertise in these rare conditions. These reference centers function as central relay station of a network of care takers, National Adrenal Networks and Endo-ERN.

## Author Contributions

KC, CA, NB, and AP contributed to the review conception, outline and design. The theoretical framework was discussed and agreed upon by all authors. Literature selection and collection was performed by KC and CA in collaboration with a trained librarian. The original focus group data were collected and analyzed by CA. The first draft of the manuscript was written by KC and CA. All authors contributed to the article and approved the submitted version.

## Conflict of Interest

The authors declare that the research was conducted in the absence of any commercial or financial relationships that could be construed as a potential conflict of interest.
